# Weakly Correlated Knowledge Integration for Few-shot Image Classification

**DOI:** 10.1007/s11633-022-1320-9

**Published:** 2022-01-21

**Authors:** Chun Yang, Chang Liu, Xu-Cheng Yin

**Affiliations:** Department of Computer Science and Technology, University of Science and Technology Beijing, Beijing, 100083 China

**Keywords:** Computer vision, pattern recognition, knowledge refinement and reuse, neural networks, machine vision

## Abstract

Various few-shot image classification methods indicate that transferring knowledge from other sources can improve the accuracy of the classification. However, most of these methods work with one single source or use only closely correlated knowledge sources. In this paper, we propose a novel weakly correlated knowledge integration (WCKI) framework to address these issues. More specifically, we propose a unified knowledge graph (UKG) to integrate knowledge transferred from different sources (i.e., visual domain and textual domain). Moreover, a graph attention module is proposed to sample the subgraph from the UKG with low complexity. To avoid explicitly aligning the visual features to the potentially biased and weakly correlated knowledge space, we sample a task-specific subgraph from UKG and append it as latent variables. Our framework demonstrates significant improvements on multiple few-shot image classification datasets.

### 1 Introduction

Deep learning approaches have achieved impressive performance on image classification tasks recently. However, most of these approaches need huge data for training. Furthermore, they are hard to be adopted to perform classification on samples from unseen classes with a limited number of examples. The challenges of learning with limited labeled data can be categorized into the few-shot learning problem. Due to the fact that annotated data can be expensive to obtain, this challenge is gaining more attention from the automation community[1–4]. In this paper, we study the popular $N$-way $K$-shot image classification task among the few-shot learning problems. Many methods introduce external knowledge to address the problem of insufficient samples, most of which adopt textual domain knowledge from label descriptions[5–10]. In particular, some works (e.g., CADA-VAE[6], Soravit's method[7], and ReViSE[8]) align the features from the visual feature domain to the textual feature domain. Many of these methods intend to work on datasets (e.g., animal with annotation[11] and CUB[12]) that provide highly correlated and structural textual descriptions. However, few such methods apply to datasets that only provide weakly correlated descriptions, e.g., the Mini-ImageNet and Tiered-ImageNet datasets. In these datasets, the label descriptions are not strongly correlated with the visual properties of the corresponding classes. It is shown in Fig. 1.

Other methods like LSFS[9] and MNE[13] exploit other information from different perspectives. For example, MNE[13] exploits information on the training set by keeping an episodic memory and fetches $K$ nearest neighbors (KNN) to extend each sample in a task. LSFS[9] uses a hierarchical structure provided by the datasets. Here, LSFS still requires the dataset to provide an extra hierarchical annotation of different classes, while MNE does not utilize information in the label description. Integrating weakly correlated knowledge from different domain sources is still an open problem in the literature.

In this paper, we propose a weakly correlated knowledge integration (WCKI) framework which can leverage nonstructural and weakly correlated knowledge extracted from different sources (i.e., visual domain and textual domain) to improve the few-shot classification performance. An overview of our framework is shown in Fig. 2.

First, we propose a unified knowledge graph, which allows the integration of knowledge transferred from different domains. Distinctive to MNE[13] that models knowledge on the training set with a memory module with hard-wired updating policy, the unified knowledge graph allows end-to-end optimization. Also different from [14,

Research Article
Manuscript received July 10, 2021; accepted November 2, 2021
Recommended by Associate Editor Ming-Ming Cheng
Colored figures are available in the online version at https://link.springer.com/journal/11633
*These authors contribute equally to this work
© The Author(s) 2022

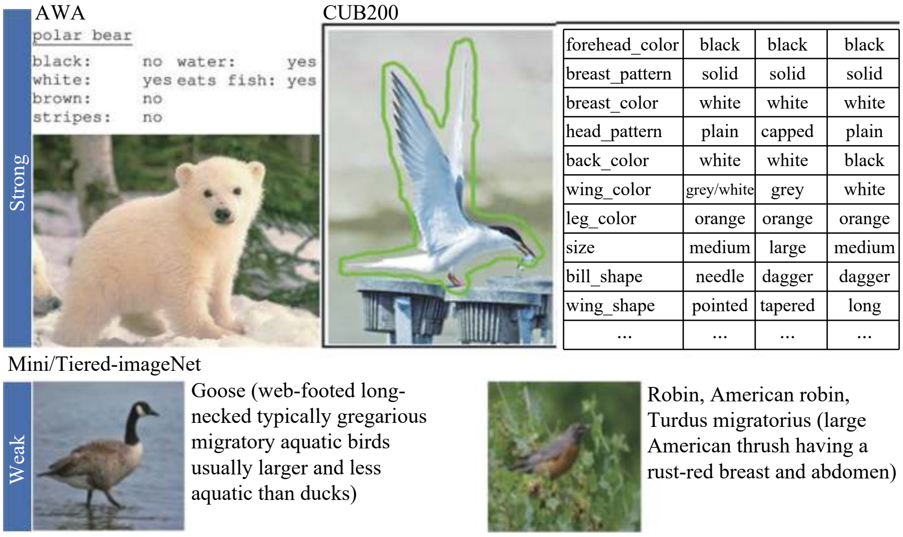

Fig. 1 Differences in annotations of different datasets. Top: Sample from AWA2 dataset (Figure adapted from [11]) and CUB dataset (Figure adapted from [12]), where the information in the description is structural and highly correlated to visual appearance. Bottom: Samples from Mini-ImageNet with WordNet annotations, where the description is less correlated to the visual properties of the corresponding object.

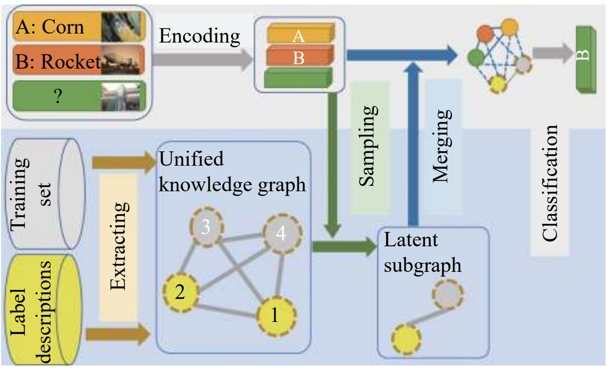

Fig. 2 Overview of our framework. In our framework, knowledge extracted from different domains is modeled with the united knowledge graph. For each specific task, we sample an "optimal" subgraph. We then "merge" them with encoded sample features and use them as latent variables for the classifier to improve accuracy.

15], our method can integrate knowledge transferred from multiple domains. In this work, we adopt two commonly used knowledge domains: textual domain knowledge[16] and visual domain knowledge collected from historical training episodes[13, 14]. Since the training set mainly consists of images from the visual domain and the model is trained to align the visual features of samples from the same classes, such knowledge is considered as visual domain knowledge.

Second, our model utilizes a differential graph attention module to sample more "relevant" knowledge proved to be able to improve both accuracy and efficiency. This module helps to reduce the computational complexity and improve the task-relevancy of the transferred knowledge. Different from the rule-based KNN approach in MNE, our graph attention module is differentiable and thus trainable, leading to a fully end-to-end trainable framework.

Finally, we take the transferred knowledge as latent variables in our framework like MNE[11], ARML[12], and [17] to avoid aligning explicitly sample features and weakly correlated transferred knowledge.

The contributions of this work are summarized as follows:

1) Proposing a weakly correlated knowledge integration framework which can transfer knowledge from multiple potentially biased sources to improve few-shot image classification task.

2) Proposing a unified knowledge graph to represent and index transferred knowledge adaptively for each specific task.

3) Proposing a graph attention module for adaptively sampling transferred knowledge for each specific task to reduce computing complexity and improve the task-relevancy of knowledge.

The source code of this paper is released at: https://www.dropbox.com/s/2ffd1dh6xyf3xzp/wcki-eval.tar.gz?dl=0.

### 2 Related works

The $N$-way $K$-shot problem is a commonly researched problem in the few-show learning field. In this problem, the model is supposed to produce label predictions for each sample as output. More specifically, $S$ contains $N\times K$ labeled samples from $N$ classes ($K$ per class). $Q$ contains $N\times K_q$ samples drawn from the training set and $K_q$ samples for each class in $S$. $K_q$ indicates the number of queries sampled for testing for each class. During the evaluation stage, a number of evaluation tasks are sampled from the testing set, and the average accuracy is used to measure the model performance. Unlike typical deep learning tasks, each contains $N \times K$ support samples that can be used to fine-tune the model for the corresponding task. An extra training set is provided to train the model used in the evaluation process. Note that the label set of the extra training set is always disjoint to the label set of the testing set. In other words, the testing set only contains samples of unseen labels in the training set.

Graph-based methods such as [18−20] are widely applied in few-shot learning for better modeling inter-class relations to relieve the data insufficient problem. These methods put support samples and query samples into one graph and inference the relation between query samples and support samples by processing the graph with a graph neural network. More specifically, TPN[20] propagates labels of each support sample to each query sample with a graph network. FGNN[18] and EGNN[19] model the input samples as graph nodes and the pairwise similarities by edges. The graph is updated by a graph network, and classification results are derived according to query-to-support edges. In this work, we adopt the second approach following EGNN[19].

However, these approaches still face the challenge of insufficient information on novel classes. Different methods are proposed to transfer and utilize external knowledge to provide more information on unseen classes. Methods like [21] use Siamese networks that transfer knowledge from another potentially biased data source. Alternatively, methods like [8, 16] exploit semantic information of class labels. However, most of such methods explicitly align the semantic embedding of the text description of the label with visual features. The performance of these approaches highly depends on the quality of label description[8] and the language model used to generate semantic embedding. To address the problem, AM3[15], learns a "convex combination" that acts as a gate to filter out potentially biased textual domain knowledge.

Other methods, on the other hand, like MNE[13], Castle[22], ARML[14], and [17] propose adopting knowledge extracted from the extra training set instead of the textual domain to enhance the classification performance. These methods use transferred knowledge as latent variables instead of aligning it with visual features of input samples, which provide some extend of robustness against bias and noise. This latent variable approach also requires no explicit correspondence between transferred knowledge data and novel classes. However, they utilize only one knowledge source and do not exploit the information of the labels. In this work, we propose a method combining two commonly used sources and adaptively sample the most relevant knowledge for each specific task.

## 3 Main method

### 3.1 Framework

In this paper, we propose a weakly correlated knowledge integration framework, as shown in Fig. 3. The proposed framework aims to alleviate the sample insufficiency by utilizing knowledge from weakly correlated sources. In the framework, the unified knowledge graph $G_k$ is proposed to adaptively integrate knowledge from different sources. In order to avoid introducing bias into the transferred knowledge, the transferred knowledge is used as latent variables instead of alignment references[16]. Further, a graph attention module is proposed, which adaptively samples a task-specific subgraph from $G_k$ to improve the relevance of the latent variable. More specifically, for each few-shot classification task, the encoder first encodes each image sample into a feature vector with a standard four-layer CNN[19, 23, 24]. Next, the observation graph $G_{obs}$ is constructed based on the embedding of each sample. The graph attention module then samples a task-

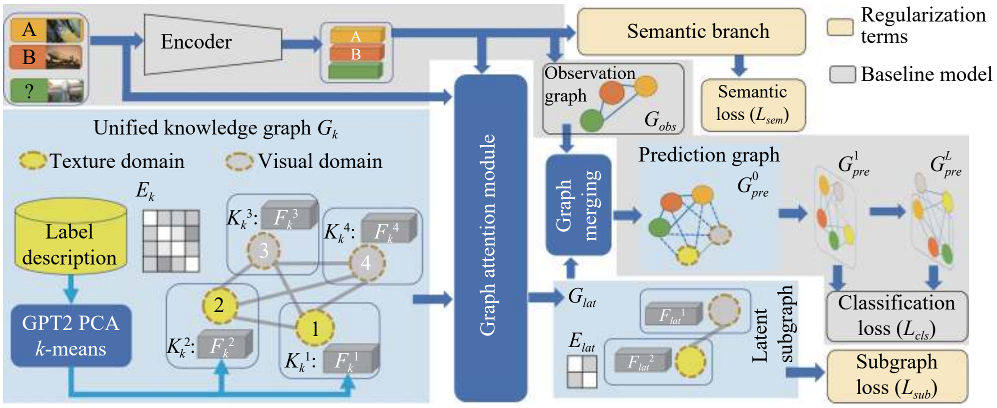

Fig. 3 Pipeline of our method. Our framework integrates knowledge extracted from weakly correlated domains with a unified knowledge graph $G_k$ and adaptively uses a more relevant "subgrap" $G_{lat}$ as latent variables according to one specific classification task. More specifically, the input data is first encoded with a CNN encoder. Then, a latent subgraph $G_{lat}$ is sampled from $G_k$ for each specific task for better task relevancy. $G_{lat}$ is merged into the observation graph $G_{obs}$, which is constructed according to the encoded labels and features. The combined graph $G_{pre}$ is updated with a multi-layer GNN.

relevant latent subgraph $G_{lat}$ from the unified knowledge graph $G_k$ according to the labels and embeddings from the samples of the supporting set. We then merge $G_{lat}$ and the observation graph $G_{obs}$ into the initial prediction $G^0{}_{pre}$. Like EGNN[19], $G^0{}_{pre}$ is iteratively updated with a multi-layer edge-feature GNN. The prediction result is obtained according to the updated edges of $G^L{}_{pre}$. Like existing methods [18, 19], we model each sample as graph nodes. The similarities between samples are modeled as the edges of the graph. Query samples are classified according to their overall similarity to each class, which is computed by averaging the similarity of all support samples in the class. Key notations in this section and algorithm block diagrams of our framework are summarized in Appendix A.

### 3.2 Unified knowledge graph

The unified knowledge graph is proposed to integrate and model the knowledge transferred from potentially biased sources. In this paper, we adopt and integrate the knowledge extracted from the training set[13] and the glossary of all nouns in WordNet[16]. These two domains form two disjoint subgraphs of $G_k$. To keep it concise, we denote these two sources as the visual domain knowledge and textual domain knowledge, respectively.

First, for the visual domain knowledge, slightly different from the memory module in MNE[13], we use a trainable graph of $N_{vis}$ nodes shared among all training tasks, avoiding the non-differentiable procedure of updating the memory entries. Since the graph is shared and optimized for all training tasks, it can be interpreted as knowledge that provides information for classification tasks on training classes. As the training tasks mainly consist of visual information, this part is considered visual domain knowledge.

Second, we use another graph of $N_{nlp}$ nodes to model the textual domain knowledge extracted from WordNet glossaries. In more detail, we first use the GPT2 model to encode the label description into word vectors, which are then averaged into the corresponding label embedding. Since the semantic embedding is extracted with a model trained on more data, we use the principal component decomposition (PCA) approach to project the semantic embedding to node features. Finally, we adopt a $k$-means clustering algorithm to reduce the number of nodes, where each center corresponds to a node in the graph. Since the textual domain model is trained on a much larger dataset, corresponding node features are locked during the training process.

Formally, the unified knowledge graph $G_k$ ($F_k$, $K_k$, $E_k$) is a graph with $N_k = N_{vis} + N_{nlp}$ nodes. Each node $i$ in $G_k$ is represented with the feature $F_k(i) \in \mathbf{R}^{C_f}$ and indexed by key $K_k(i) \in \mathbf{R}^{C_k}$. $C_f$ and $C_k$ are channel numbers of node features $F_k$ and keys $K_k$, respectively. The keys $K_k$ for all nodes are random initialized trainable tensors. The edges $E_k$ are initialized according to the cosine distance between $F_k(i)$ and $F_k(j)$.

### 3.3 Graph attention module

The graph attention module shown in Fig. 4 is proposed to improve the relevance of the transferred knowledge by sampling a more relevant part for each specific task. This process also reduces the computational complexity by reducing the total number of nodes in the graph. More specifically, the module first encodes support samples and labels into task representation with the projector module, then samples a task-specific latent subgraph $G_{lat}$ from $G_k$ according to the task representation. Then, the graph sampler samples $G_{lat}$ from $G_k$ according to the queries.

The projector module first summarizes textual features from support sample labels $L_t$ and support image embedding $S_t$ into the task feature $T_t$. It then decodes $T_t$ into queries $Q_t$ for nodes in $G_{lat}$, where each query $Q_t(i)$ corresponds to a sampled node in $G_{lat}$, i.e., $Q_t(i)$ would be the query for the $i$-th node in $G_{lat}$. To generate task feature $T_t$, we first use the visual feature encoder $T_s$ and the textual encoder $T_l$ to encode the corresponding label information. Then, we sum these two types of extracted information into a feature $T_t$, which represents the fea-

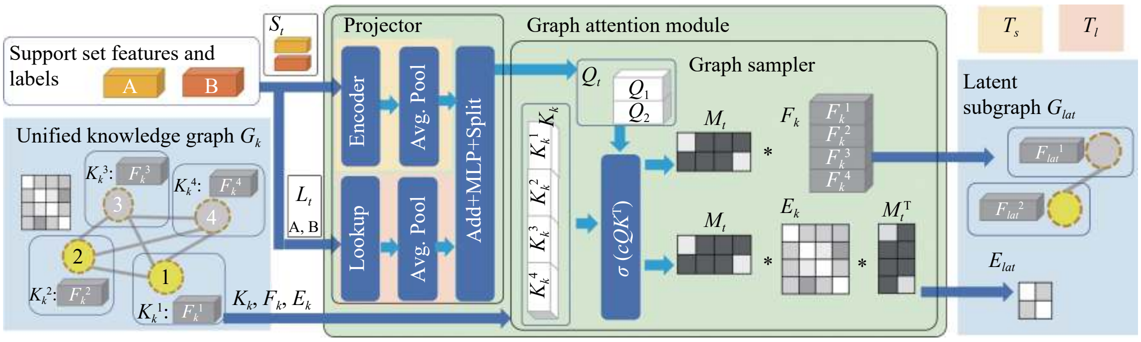

Fig. 4 Illustration of graph attention module. We first build the representation for each task by encoding feature from support samples and their labels into the task feature $T_t$ using encoder $T_s$ and $T_l$, respectively. $T_t$ is then decoded into queries $Q_t$ for nodes in the subgraph. Then, a task-specific latent subgraph $G_{lat}$ is obtained by sampling nodes and corresponding edges from the unified knowledge graph according to $Q_t$.

ture of a specific task:

$$T_t = \lambda_v T_s(S_t) + \lambda_l T_l(L_t). \quad (1)$$

The function $T_s$ encodes the visual features of all support samples in the task. Here, function $T_s$ first projects the feature vector of each sample from the support set into a latent space with a multi-layer perception (MLP), then averages all projected features into the visual "summary" of the task. Function $T_l$ encodes the label information of tasks: We first look up the corresponding nodes of the labels and then project the associated features into the summary space with another MLP and average them as the textual "summary".

To sample a task-specific subgraph from $G_k$, we use $N_{lat}$ different decoders to decode them into $N_{lat}$ queries, one corresponding to a node in the latent subgraph $G_{lat}$. The $G_{lat}$ is sampled by applying attention on $G_k$ with $Q_t$ as the query, $K_k$ as key, and $F_k$, $E_k$ as values, i.e.,

$$\begin{aligned}
&Q_t(i) = Dec_i(T_t)\\
&A = cQ_t K_k^{\mathrm{T}}\\
&M_t(i,j) = \frac{\mathrm{e}^{A(i,j)}}{\sum_j^{N_k} \mathrm{e}^{A(i,j)}}\\
&F_{lat} = M_t F_k\\
&E_{lat} = M_t \delta(E_k) M_t^{\mathrm{T}}
\end{aligned} \quad (2)$$

where $c$ is a constant factor that controls the tendency towards one-hot, and $\delta$ is the sigmoid function that restricts the range of elements in $E_k$.

We merge the latent subgraph $G_{lat}$ and the observed graph $G_{obs}$ into the initial prediction graph $G^0{}_{pre}$ via the following process. We first concentrate on the node features of $G_{obs}$ and $G_{lat}$, formally:

$$F_{pre}^0 = F_{obs} \,||\, F_{lat} \quad (3)$$

where $||$ is the concatenation operator. For edges, we keep the edges between $G_{obs}$ and $G_{lat}$, and fill the missing edges with 0.5, i.e.,

$$E_{pre}^0 = \begin{pmatrix} E_{obs} & 0.5I \\ 0.5I & E_{lat} \end{pmatrix} \quad (4)$$

where $I$ is the unit matrix, and all elements are ones. 0.5 is the value that indicates unknown similarity in EGNN[19].

In our graph attention module, $G_{lat}$ will be a "relaxed subgraph" of $G_k$ and mathematically one subgraph of $G_k$ when the following three conditions are met. First, $M_t$ is a binary matrix, i.e.,

$$M_t(i,j) \in \{0,1\}. \quad (5)$$

Second, a node in the latent subgraph should consist of one and only one node in the original graph,

$$\sum_j^{N_k} M_t(i,j) = 1 \quad (6)$$

where $N_k$ is the size of the unified knowledge graph $G_k$.

The third condition is that the united knowledge graph nodes should be either sampled only once or not sampled. When (5) holds, this condition reduces to that nodes in $G_k$ shall not be sampled more than once, i.e.,

$$\sum_i^{N_{lat}} M_t(i,j) \le 1. \quad (7)$$

To make the subgraph sampling process differential, our method (2) relaxes the above conditions, making our "subgraph" a generalized case of the subgraph in terms of discrete math. Moreover, since we use softmax activation on the rows of $M_t$, condition two, i.e., (6), always holds.

Condition one, i.e., (5), will not be strictly met, but can be considered approximately met because the rows of $M_t$ will tend to be one-hot. This tendency is due to the gradient property of the softmax function $\sigma$. To simplify the representation, we discuss $\frac{\partial\sigma(x)(i)}{\partial x(i)}$ and $\frac{\partial\sigma(x)(t\neq i)}{\partial x(i)}$ separately, i.e.,

$$\begin{aligned}
&\frac{\partial\sigma(x)(i)}{\partial x(i)} = \frac{\mathrm{e}^{x(i)}\sum_j^N \mathrm{e}^{x(j)} - \mathrm{e}^{2x(i)}}{\sum_j^N \mathrm{e}^{x(j)} \sum_j^N \mathrm{e}^{x(j)}} = \\
&\qquad \sigma(x)(i) \times (1-\sigma(x)(i)).\\
&\frac{\partial\sigma(x)(t\neq i)}{\partial x(i)} = -\frac{\mathrm{e}^{x(t)}\mathrm{e}^{x(i)}}{\sum_j^N \mathrm{e}^{x(j)} \sum_j^N \mathrm{e}^{x(j)}} = \\
&\qquad -\sigma(x)(t)\,\sigma(x)(i).
\end{aligned} \quad (8)$$

The gradient of the softmax function is close to 0 when its output is close to one-hot, i.e., $\max(\sigma(x)(i))$ is close to 1, and the other terms are consequently close to 0.

For condition three, we use a regularization term to reduce the pairwise-node similarity of the subgraph to enforce a close approximation. Having repeated nodes in $G_{lat}$ is the only case where condition three is violated, while conditions one and two are satisfied. To avoid this situation, we add a regularization term to enlarge the pairwise distance among node features in $G_{lat}$. Thus, we consider $G_{lat}$ as a generalized subgraph of $G_k$ by relaxing conditions one and three slightly.

### 3.4 Optimization

In order to improve the efficiency of the model, we add a regularization term $L_{sub}$ in the module. $L_{sub}$ is applied to increase the diversity of nodes in $G_{lat}$. We enforce the diversity by increasing the pairwise cosine distance of node features, i.e.,

$$L_{sub} = \frac{1}{N_{lat}N_{lat}} \sum_{i}^{N_{lat}} \sum_{j}^{N_{lat}} \frac{(F_{lat}(i))^{\mathrm{T}} F_{lat}(j)}{|F_{lat}(i)| \times |F_{lat}(j)| + \varepsilon}. \quad (9)$$

This regularization term also helps to enforce condition three of (6) for the latent subgraph $G_{lat}$ to be close to a strict subgraph of $G_k$. This is because repetitive nodes will lead to larger losses due to the fact that identical vectors always have the largest cosine similarity, i.e.,

$$\cos(F_a, F_b) \leq \cos(F_a, F_a) = 1. \quad (10)$$

We adopt the edge classification loss $L_{cls}$ in EGNN[19] for classification and apply it to each layer of the graph neuro network, i.e.,

$$L_{cls} = \sum_{i=1}^{T} w_i BCE(E_{pre}^{(i)}, E^*). \quad (11)$$

Note that edges connected to latent nodes do not contribute to the classification loss, and $w$ is set to [0.5, 0.5, 1] following [19].

We also adopt a semantic branch[25] to further improve the performance. Slightly different from the original work, our implementation performs classification on all labels in a batch instead of the whole training set to reduce computation. The module takes the label and visual features as input and produces a classification loss $L_{sem}$.

The final objective function is the weighted sum of the classification loss $L_{cls}$, the semantic branch loss $L_{sem}$, and the subgraph loss $L_{sub}$, i.e.,

$$L = L_{cls} + \lambda_{sub} L_{sub} + \lambda_{sem} L_{sem}. \quad (12)$$

In our experiment, $\lambda_{sub}$ and $\lambda_{sem}$ are empirically set to 0.1 as these two are regularization terms, hence less important than $L_{cls}$.

## 4 Experiments

### 4.1 Datasets

In this section, we use three datasets to validate the proposed framework. More specifically, we use Mini-ImageNet and Tiered-ImageNet, which provide less visual-correlated label annotation. Mini-ImageNet is a subset of ImageNet with 100 classes, consisting of 600 images per class. The dataset is split into the training set (64 classes), the validation set (16 classes), and the testing set (20 classes)[26]. Tiered-ImageNet is also a subset of ImageNet with 608 classes, and each class contains 600 images. Different from Mini-ImageNet, the Tiered-ImageNet dataset has structural information in label annotation. The classes are categorized into 34 more general classes. The splitting of this dataset is also based on the general classes. The training set has 20 classes, the validation set has 6 general classes, and the testing set has 8 classes. We also use the CUB-2011 dataset that provides detailed annotations closely related to visual traits. CUB-2011 contains images of 200 different bird species. The dataset is split into the training set (100 classes), the validation set (50 classes), and the testing set (50 classes)[12].

### 4.2 Implementation details

Our implementation is based on the EGNN[19] code base, which uses the Pytorch framework. For comparison with the latest methods, we also trained a heavier model on Mini-ImageNet using the pre-trained ResNet12 backbone from FEAT[27]. We locked the weight of the pre-trained ResNet12 backbone to prevent overfitting.

We adopt two popular protocols used in the evaluation. For the first protocol, 600 random tasks are sampled from the testing set, where each task contains 15 query samples per class. We also adopt the one query protocol[28], where only one query image is used for each class in a task. In this protocol, we sample 50 000 queries in 10 000 tasks to evaluate the performance of our model. For both protocols, the average accuracy of all evaluation tasks is used as a performance metric.

During training, we train our framework for 100 000 iterations on the training set for all three datasets. We use the Adam solver[29], and the learning rate is initially set to $10^{-3}$. The learning rate is set to decay by a half for every 15 000 iterations for Mini-ImageNet and 30 000 for Tiered-ImageNet. We validate the model on the validation set for every 5 000 iterations and select the best model for testing. The batch size is set to 18 due to the limit of hardware resources. For the CUB dataset, we adopt the setup of Tiered-ImageNet with different batch sizes according to available hardware resources.

### 4.3 Ablation study

For simplicity, we use the second training and evaluation protocol (using one query per class) in this section. We perform ablation studies to each module baseline method. The experimental results of the ablation study on the Mini-ImageNet dataset are shown in Table 1. In Table 1, GAM denotes the graph attention module. "Textual" indicates knowledge transferred from the label description, and "Visual" indicates knowledge transferred from the training set. We use base to indicate the baseline method, and the other experiments are named with three characters following the specified rule: The first character indicates knowledge domains, the second indicates different graph sampler configs, and the third indicates whether the semantic branch is used.

The baseline method, indicated with gray background in Fig. 3, made a few implementation changes to make it

Table 1 Ablation study of the proposed framework

| Experiment | Visual | Textual | GAM | $L_{sem}$ | Accuracy (%) | Standard deviation (%) |
|---|---|---|---|---|---|---|
| Base | | | | | 60.15 | 8.32 |
| A00 | √ | √ | | | 61.02 | 8.29 |
| VV0 | √ | | Visual-only | | 61.01 | 8.39 |
| AV0 | √ | √ | Visual-only | | 61.30 | 8.44 |
| AA0 | √ | √ | Visual+Textual | | 61.64 | 8.66 |
| VAD | √ | | Visual+Textual | √ | 62.64 | 8.28 |
| AAD | √ | √ | Visual+Textual | √ | 62.93 | 8.62 |

compatible with our modules. The open-source EGNN code produces the same results as our baseline when trained with the same dataloader[1] and hyper-parameters.

Comparing base and A00, we observe that using transferred knowledge as latent variables can benefit classification performance. Experiments of VV0 VS. AV0, VAD VS. AAD validate the effectiveness of the textual domain in the unified knowledge graph. Comparing A00 and AV0 shows the effectiveness of the graph attention module, comparing AV0 and AA0 shows the effectiveness of introducing textual information into the graph attention module.

To sum up, each proposed module effectively increases the classification accuracy, and the whole method shows a significant improvement against the baseline method by improving the accuracy on most classes. The improvement can be seen in the intuitive comparison of tasks sampled from the testing set in Fig. 5. Statistical changes for each class are shown in Fig. 6. This result indicates that our model is generally effective for most classes. We visualize the difference between our method

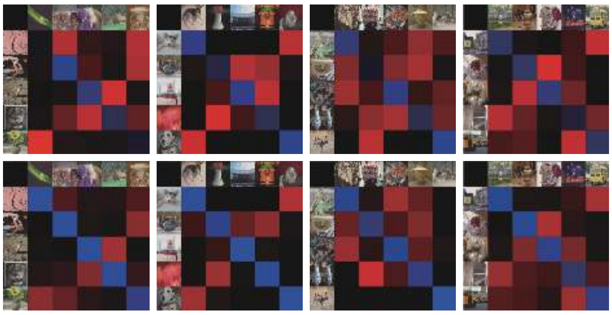

Fig. 5 Prediction results on tasks sampled from the training set. The top row shows the results from the baseline, while the bottom row shows the results of our method on the same tasks. In each task, the predicted similarity between samples (ranging from 0 to 1) is shown in color intensity, where zero similarity leads to a black square. Here, blue means "correct" response, and red indicates "wrong" responses. Pictures on the top row are query samples, while those on the left-most column are support labels.

[1] https://github.com/khy0809/fewshot-egnn/issues and the baseline method. To break down the improvement shown in Fig. 6 into more details, we then visualize the delta between the confusion matrices of the baseline method and our method in Fig. 7. The delta suggests that the improvement comes primarily from the misclassification among different clusters. The evidence is that most non-diagonal squares (indicating misclassification across clusters) are dominated by purple cells. This observation suggests that our method is able to utilize textual domain knowledge to improve discrimination between most "general" classes ($k$-means centers).

Another observation is that our model slightly increased the misclassification between cluster 4 (dogs) and cluster 5 (mostly large mammals). This is likely due to that these two clusters are semantically very close to each other. Note that the only two classes with a performance drop come from these two clusters. This shows that the

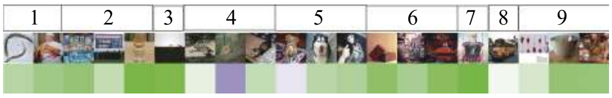

Fig. 6 Change of classification accuracy for each class on the testing set. Green indicates our methods show accuracy improvement against the base method, and purple indicates dropping accuracy. Color intensity indicates the extent of accuracy change. The numbers in the top ribbon indicate the cluster ID in the $k$-means algorithm.

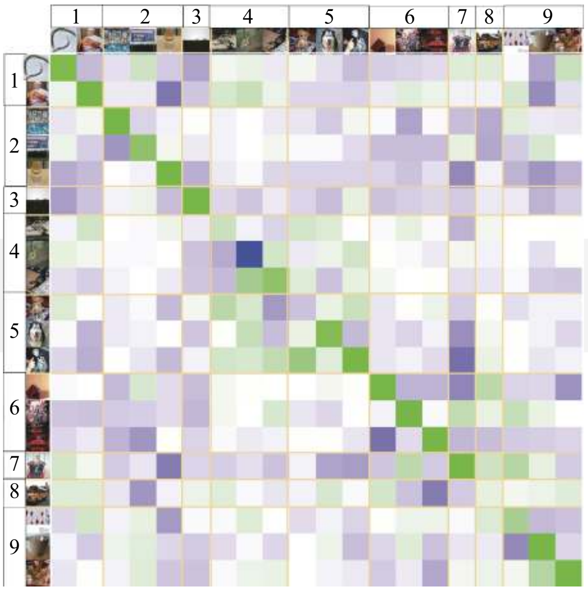

Fig. 7 Visualization of the differences of confusion matrices of the baseline and our method. Ground truth labels are shown in the top row, and prediction labels are shown in the left column. Grid $(i, j)$ indicates the number of cases where class $i$ is recognized as class $j$. Green indicates the case with an increasing occurrence in our method, and purple indicates a decreasing case number. Color intensity indicates the absolute value of the number of differences. Labels from different clusters are split by yellow lines. Note that some of the diagonal elements are clipped for better visibility. The unclipped diagonal cells are shown in Fig. 5.

"noise" in the textual domain may not be completely avoided, which is also a future topic for our research. It is also noticed that a large margin between validation and testing sets can be identified, mostly because the testing set has a more skewed data distribution in both textual and visual domains. We will also leave this as a future topic.

#### 4.4 Few-shot classification

We perform experiments mainly on two different datasets: Mini-ImageNet and Tiered-ImageNet. Following the conventional way[13, 24], we train and evaluate our method with both 5-way 1-shot setup and 5-way 5-shot setup. We also train our model both with and without the transduction, which allows us to offer a fair comparison to popular methods. Transduction[20] indicates that the relations among testing samples are exploited. Methods known to be using such tricks will be marked with "(BN)" or "(T)", where "(BN)" means the model uses the task statistic[19] during evaluation, and "(T)" means more sophisticated approaches are applied. Methods involving transduction can be sensitive to the total number of queries in each task. Therefore, we also list this factor in corresponding tables. We also observe that some works may have different performance with different reimplementation and training/evaluation policies. In such cases, we will use the results from the original paper if not specified.

On the Mini-ImageNet dataset, our method shows competitive performance with and without transduction. Our model also shows competitive performance with many popular few-shot classification methods. The results are shown in Table 2, as the evaluation protocol and training methods may vary between different works, we give more details on the comparisons of results. More specifically, we list the number of queries in each task, the backbone used, and whether or not the validation set is used for each method. Note that the number of queries per class only affects the transduction setup. The performance of our method produces better performance than many state-of-the-art methods in transduction settings. Our method also produces promising results on non-transduction settings in both 5-way 1-shot and 5-way 5-shot problems. Particularly, our method generally leads to an improvement of 2%–3% against the EGNN[19] on accuracy in all setups.

On the Tiered-ImageNet dataset, we perform experiments using the transductive setups (QPC = 1 and QPC = 15), and the results are listed in Table 3. Our method also demonstrates promising performance compared to many popular methods. In more detail, our method enjoys better performance with the 1-shot setup with both protocols, i.e., around 4% higher than the TPN[20]. On the 5-shot setup, our method's accuracy is slightly lower than the TPN method[20] by 0.3%. The results indicate that our framework can effectively utilize the transferred knowledge.

We also conduct experiments on the CUB-2011 dataset to validate the generalization ability on fine-grained classification tasks. Our model demonstrates competitive performance (presented in Table 4) with a simple Conv4 backbone. Our method outperforms Antreas's method[42] by 10% in terms of accuracy on 1-shot tasks. In 5-shot tasks, our method again achieved a 6% improvement in accuracy. This is possibly due to the better correlation between the annotation and the visual features. These experiments demonstrate the effectiveness of our proposed framework in few-shot image classification tasks on different datasets and with different protocols. Our framework obtains reasonably good performance with the transductive evaluation and attains promising performance without transduction where information among queries can be exploited. These results validate that the proposed framework can utilize weakly correlated knowledge from different sources (e.g., the visual domain and the textual domain) to reach promising and robust performance on different datasets.

### 5 Discussions

#### 5.1 Computational complexity

Our proposed framework is not significantly larger than the baseline EGNN model in terms of computational complexity. The extra cost is brought by two parts: the size incremental of graph $G_{pre}$ caused by the auxiliary latent subgraph and the newly introduced graph attention module. Intuitively, the second part is not much large since the latent subgraph is generally small, as we control the size of the latent subgraph $G_{lat}$ with the proposed graph attention module to avoid huge graphs for GNN. As for the graph attention module, the projector is a network much smaller than the encoder, and the graph sampler also has low complexity.

In more details, the addition computation complexity is derived as following: For the additional cost caused by $G_{pre}$, the complexity is changed from

$$O\left(|G_{obs}|^2 C_f\right) \tag{13}$$

to

$$O\left(\left(|G_{obs}| + |G_{lat}|\right)^2 C_f\right) \tag{14}$$

and the delta (the difference of (14) and (13)) is

$$\begin{aligned} &O\left(\left(|G_{lat}|\right)^2 C_f\right) + 2O\left(|G_{lat}| \times |G_{obs}|\, C_f\right) = \\ &O\left(|G_{lat}| \times |G_{obs}|\, C_f\right). \end{aligned} \tag{15}$$

Because $|G_{lat}|$ is always smaller than $|G_{obs}|$ in this paper, the extra complexity in this module is just a constant factor less than 3.

For the graph attention module, the projector is much smaller compared to the CNN encoder. Therefore, we fo-

Table 2 Comparative results on Mini-ImageNet. The method * indicates results from code and model released by the authors of EGNN[19].

| Method | Venue | Backbone | Train with val | Query per class | Accuracy (%) | |
|---|---|---|---|---|---|---|
| | | | | | $N$=5, $K$=1 | $N$=5, $K$=5 |
| Matching network[30] | NIPS 16 | Conv4 | Yes | / | 46.6 | 60.0 |
| Reptile[28] | CoRR 18 | Conv4 | – | / | 47.07 | 62.74 |
| IMP[31] | ICML 19 | Conv4 | No | / | 49.2 | 64.7 |
| Prototypical net[32] | NIPS 17 | Conv4 | Yes | / | 49.42 | 68.20 |
| ARML[14] | ICLR 20 | Conv4 | No | / | 49.2 | 64.3 |
| Relation network[33] | CVPR 18 | InceptionV2 | No | / | 50.44 | 65.32 |
| GNN[18] | ICLR 18 | Conv4 | No | / | 50.33 | 66.41 |
| R2D2[34] | ICLR 19 | Conv4 | No | / | 51.8 | 68.4 |
| SAML[35] | ICCV 19 | Conv4 | No | / | 52.22 | 66.49 |
| GCR[36] | ICCV 19 | Conv4 | No | / | 54.61 | 71.21 |
| PARN[37] | ICCV 19 | Conv4 | No | / | 55.22 | 71.55 |
| EGNN[19] | CVPR 19 | Conv4 | No | / | 52.86 | 66.85 |
| STANet-S[25] | AAAI 19 | Conv4 | No | / | 53.11 | 67.16 |
| Ours | – | Conv4 | No | / | 55.66 | 70.28 |
| SNAIL[38] | ICLR18 | Res12 | No | / | 55.71 | 68.88 |
| AdaResNet[39] | ICML18 | Res12 | No | / | 56.88 | 71.94 |
| Ours | – | Res12 | No | / | 57.01 | 71.97 |
| Reptile (T)[28] | CoRR18 | Conv4 | No | 1 | 49.97 | 65.99 |
| EGNN(T)*[19] | CVPR19 | Conv4 | No | 1 | 59.18 | 76.37 |
| Ours (T) | – | Conv4 | No | 1 | 62.93 | 79.51 |
| Ours (T) | – | Res12 | No | 1 | 65.76 | 82.01 |
| TEWAM (T)[40] | ICCV19 | Conv4 | No | 15 | 60.07 | 63.11 |
| MAML (BN)[24] | ACL19 | Conv4 | No | 15 | 48.70 | 63.11 |
| Relation net (T)[33] | CVPR18 | Conv4 | No | 15 | 50.44 | 65.32 |
| TPN (T)[20] | ICLR19 | Conv4 | No | 15 | 53.75 | 69.43 |
| MNE (T)[13] | CoRR19 | Conv4 | No | 15 | 59.92 | 71.76 |
| Ours (T) | – | Conv4 | No | 15 | 60.65 | 72.84 |
| Ours (T) | – | Res12 | No | 15 | 61.44 | 72.21 |

cus on analyzing the graph sampler submodule. Computing $M_t$ takes the complexity:

$$O\left(|G_{lat}| \times |G_k| C_k\right) \tag{16}$$

where $C_k$ is the dimension of keys in $G_k$. Sampling nodes takes

$$O\left(|G_{lat}| \times |G_k| C_f\right) \tag{17}$$

and sampling edges takes

$$O\left(|G_{lat}| \times |G_k|^2\right). \tag{18}$$

In this work, we have a small $G_k$ due to the concern of training variance and the magnitude of the gradient in the attention module. Hence, this part is also considerably light-weighted. However, the only quadratic term of $|G_k|$ in our entire framework, which appears in (18), does not include the feature dimension, $C_f$. This property offers our framework further potential to work with a large knowledge base $G_k$ without losing the relationship $E_k$ while maintaining a reasonable speed.

### 5.2 Textual domain

Quantity results (Table 1) have shown that the textual domain provides useful information. However, intuitively the description is not always highly correlated to their visual traits, which can be observed in Fig. 1. This can also be supported by the visualization of the result of the $k$-means clustering results shown in Fig. 8. We can see that classes may or may not have intuitive common visual properties when the description vectors are se-

Table 3 Performances on Tiered-ImageNet with Conv4 backbone. QPC stands for query per class. Results marked with + are derived from TPN[20].

| Method | QPC | Accuracy (%) | |
|---|---|---|---|
| | | $N$=5, $K$=1 | $N$=5, $K$=5 |
| MAML[24] | No | 51.67 | 70.30 |
| Reptile[28] | / | 48.97 | 66.47 |
| Prototypical net[32]+ | / | 48.58 | 69.57 |
| MAML (BN)[24] | 15 | 53.23 | 70.83 |
| Relation net (BN)[33]+ | 15 | 54.48 | 71.31 |
| TPN (T)[20] | 15 | 57.53 | 72.58 |
| Ours (T) | 15 | 61.82 | 72.28 |
| Reptile (T)[28] | 1 | 52.36 | 71.03 |
| EGNN (T)[19] | 1 | – | 80.15 |
| Ours (T) | 1 | 61.45 | 80.55 |

Table 4 Performances on CUB200 with Conv4 backbone. QPC stands for query per class.

| Method | QPC | Accuracy (%) | |
|---|---|---|---|
| | | $N$=5, $K$=1 | $N$=5, $K$=5 |
| Chen et al.[41] | / | 47.12 | 64.16 |
| ARML (T)[14] | / | 52.91 | – |
| Matching network[30] | / | 61.16 | 72.80 |
| Chen et al. ++[41] | / | 60.52 | 79.34 |
| MAML (BN)[24] | 15 | 55.92 | 72.09 |
| Self-Critique (T)[42] | – | 65.56 | 77.09 |
| Ours | 15 | 75.44 | 83.67 |
| Ours | 1 | 80.59 | 92.30 |

mantically close, i.e., the samples in cluster 5 are mostly dogs, while cluster 2 contains a lot of things that are visually different. We can also observe that not all clusters are interpretable, indicating that the description and the GPT2 encoder may introduce bias. Due to these two reasons, we decided to use the transferred knowledge as latent variables rather than applying direct distance constraints.

### 6 Conclusions

To address the insufficient data problem in few-shot image classification tasks, we propose a weakly correlated knowledge integration framework. In the proposed framework, we use a unified knowledge graph to integrate knowledge from different domains into one feature space where relations among different domains are modeled with corresponding edges. The proposed attention-based graph attention module adaptively improves both the effectiveness and efficiency of our framework. The ablation studies show that each module is effective with few-shot learning tasks. Our framework also demonstrates promising results on different datasets.

### Acknowledgements

The research was supported by National Key Research and Development Program of China (No. 2020AAA09701), and National Natural Science Foundation of China (Nos. 62076024 and 62006018).

### Open Access

This article is licensed under a Creative Commons Attribution 4.0 International License, which permits use, sharing, adaptation, distribution and reproduction in any medium or format, as long as you give appropriate credit to the original author(s) and the source, provide a link to the Creative Commons licence, and indicate if changes were made.

The images or other third party material in this article are included in the article's Creative Commons li-

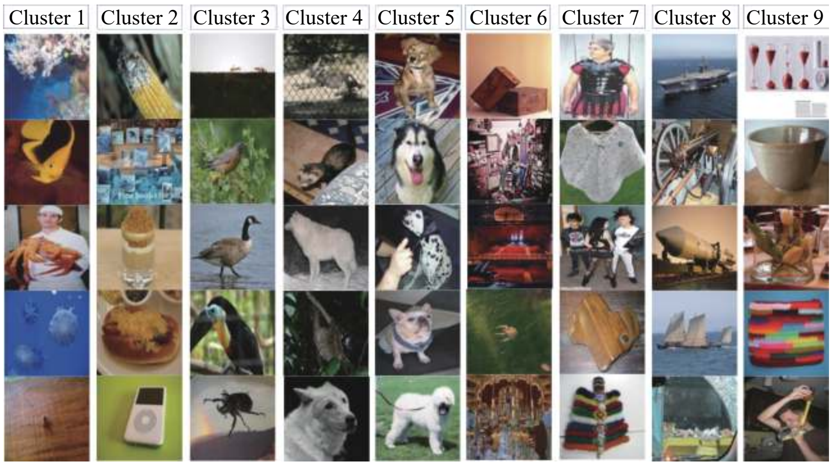

Fig. 8 Some $k$-means clustering results on the encoded description of Mini-ImageNet. Each column indicates a cluster and only five classes in each cluster are shown.

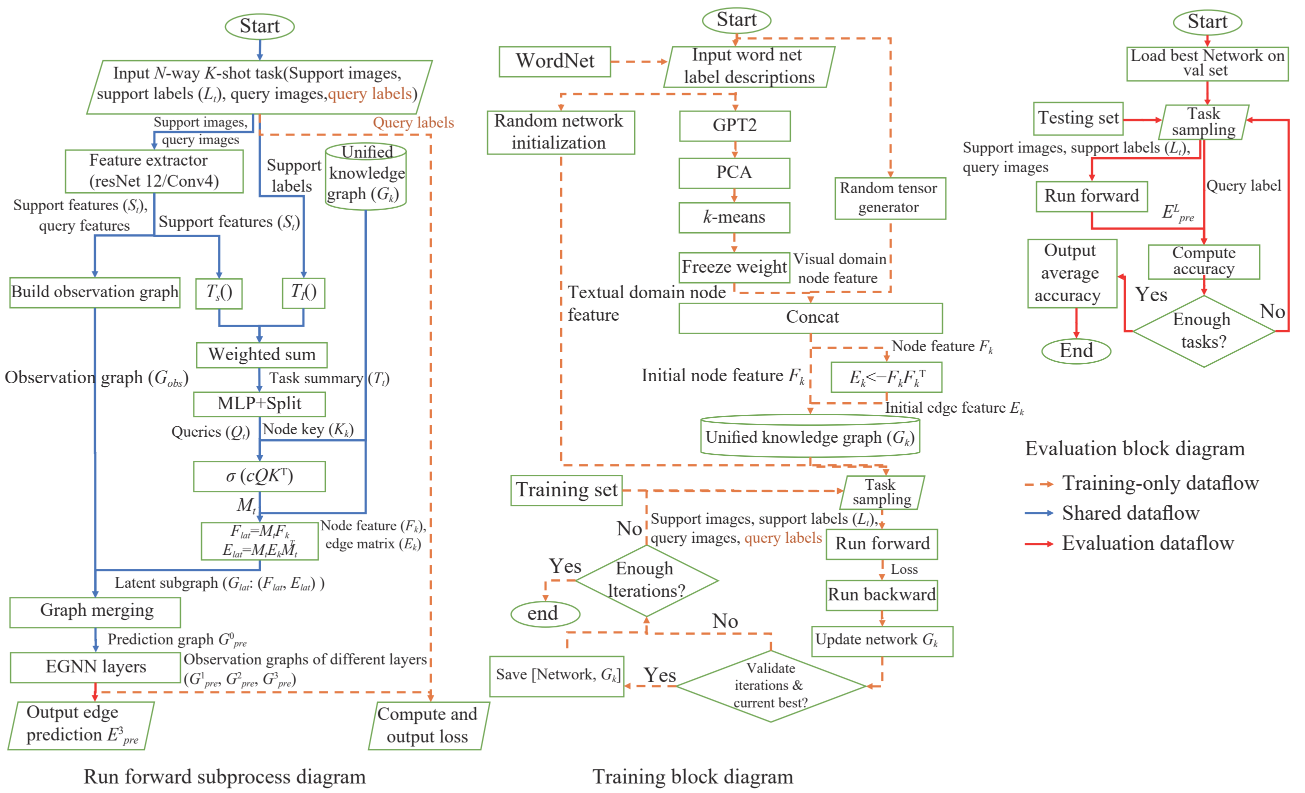

Fig. A.1 Algorithm block diagram of our framework

Table A.1 Notation table

| Notation | Defined in | Brief information |
|---|---|---|
| $G_k$ | S3 (1+) | The proposed unified knowledge graph is used to model transferred knowledge. |
| $G_{obs}$ | S3 (2+) | Graph constructed with visual features $S_t$. Most conventional GNN-based methods use this as $G_{pre}$. |
| $G^0{}_{pre}$ | S3 (2−) | Combination of $G_{lat}$ and $G_{obs}$. This graph is the input of GNN. |
| $G^i{}_{pre}$ | S3 (2+) | $G_{pre}$ after the $i$-th GNN layer. |
| $N_k$ | S3.1 (1−) | Number of nodes in $G_k$. |
| $E_k$ | S3.1 (1+) | The edges in $G_k$. |
| $K_k$ | S3.1 (1+) | Keys of nodes in $G_k$. Used as keys in the proposed graph attention module. |
| $F_k$ | S3.1 (1+) | Node features in $G_k$. Represent entries in transferred knowledge, e.g., cluster center of word vectors. |
| $G_{lat}$ | S3.1 (2) | The latent subgraph, which is an optimal subgraph of $G_k$ with regard to task $t$. Used as a latent variable in the framework. |
| $S_t$ | S3.2 (1−) | Embedding of samples in support set for task $t$, i.e., CNN features of $N \times K$ support set images in the task. |
| $L_t$ | S3.2 (1−) | Embedding of $N$ labels in task $t$, i.e., GPT features of $N$ corresponding label descriptions. |
| $T_s$ | S3.2 (1−) | Visual feature encoder, encodes $S_t$ to visual representation of the task $t$. |
| $T_l$ | S3.2 (1−) | Textual feature encoder, encodes $L_t$ to textual representation of the task $t$. |
| $T_t$ | S3.2 (1+) | "Summary" of task $t$, i.e., a combination of outputs from $T_s()$ and $T_l()$. |
| $N_{lat}$ | S3.2 (1+) | Size of the latent subgraph $G_{lat}$. |
| $Dec_i$ | S3.2 (1+) | The $i$-th decoder mapping task summary $T_t$ to query $Q_t(i)$ for the $i$-th node in $G_{lat}$ for task $t$. |
| $Q_t$ | S3.2 (1+) | Queries for $G_{lat}$ nodes for task $t$. Generated by decoding $T_t$ with $N_{lat}$ decoders. |
| $M_t$ | S3.2 (2−) | Affinity matrix between knowledge entries (nodes in $G_k$) and task $t$. |
| $F_{lat}(i)$ | S3.2 (2+) | The $i$-th node in the latent subgraph $G_{lat}$. |

cence, unless indicated otherwise in a credit line to the material. If material is not included in the article's Creative Commons licence and your intended use is not permitted by statutory regulation or exceeds the permitted use, you will need to obtain permission directly from the copyright holder.

To view a copy of this licence, visit http://creativecommons.org/licenses/by/4.0/.

### Appendix A

This paper contains many notations. To make it more clear, we list the important ones in this lookup table (Table A.1). For each notation, we list the section where it is defined. The text in the bracket may help locate it faster, e.g., S3 (1+) means the notation is defined in the latter half of the first paragraph in Section 3. We also provide the algorithm block diagrams shown in Fig. A.1 for the training and evaluation process to make the whole framework clearer.

### Appendix B

In this appendix, we provide more details on model dynamic and hyper-parameter sensitivity. For model dynamics, we show the curve of each loss term shown in Fig. B.1 of our method (AAD in Table 1) in the 5-way 1-shot training process. All loss terms drop alongside the training process. Occlusions in $L_{sem}$ and $L_{cls}$ are possibly

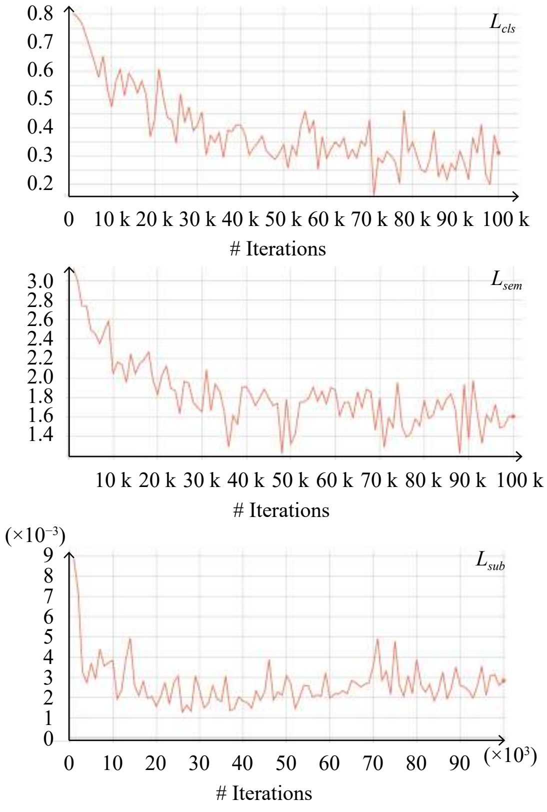

Fig. B.1 Algorithm block diagram of our framework due to the noisy ImageNet dataset. $L_{sub}$ indicates that the average pairwise distance of node features in the latent subgraph is properly controlled. The sensivity against different loss weights in (12) is also analyzed and shown in Table B.1.

Table B.1 Sensitivity against different loss weights

| Experiment | $\lambda_{sub}$ | $\lambda_{emb}$ | Accuracy (%) |
|---|---|---|---|
| Ours | 0.1 | 0.1 | 62.93 |
| 1 | 0.3 | 0.1 | 61.31 |
| 2 | 0.5 | 0.1 | 61. 95 |
| 3 | 0.1 | 0.3 | 61.46 |
| 4 | 0.1 | 0.5 | 61.37 |
| 5 | 0.3 | 0.3 | 61.53 |

## References

[1] Gu J Q, Hu H F, Li H X (2018). Local robust sparse representation for face recognition with single sample per person. IEEE/CAA Journal of Automatica Sinica.

[2] Liu D Y, Xu J, Zhang P Y, Yan Y H (2019). Investigation of knowledge transfer approaches to improve the acoustic modeling of Vietnamese ASR system. IEEE/CAA Journal of Automatica Sinica.

[3] Ohata E F, Bezerra G M, das Chagas J V S, Neto A V L, Albuquerque A B, de Albuquerque V H C, Filho P P R (2021). Automatic detection of COVID-19 infection using chest X-ray images through transfer learning. IEEE/CAA Journal of Automatica Sinica.

[4] Li Y, Xu D (2021). Skill learning for robotic insertion based on one-shot demonstration and reinforcement learning. International Journal of Automation and Computing.

[5] Xian Y Q, Akata Z, Sharma G, Nguyen Q, Hein M, Schiele B (2016). Latent embeddings for zero-shot classification. Proceedings of IEEE Conference on Computer Vision and Pattern Recognition.

[6] Schönfeld E, Ebrahimi S, Sinha S, Darrell T, Akata Z (2019). Generalized zero- and few-shot learning via aligned variational autoencoders. Proceedings of IEEE/CVF Conference on Computer Vision and Pattern Recognition.

[7] Changpinyo S, Chao W L, Gong B Q, Sha F (2016). Synthesized classifiers for zero-shot learning. Proceedings of IEEE Conference on Computer Vision and Pattern Recognition.

[8] Tsai Y H H, Huang L K, Salakhutdinov R (2017). Learning robust visual-semantic embeddings. Proceedings of IEEE International Conference on Computer Vision.

[9] Li A X, Luo T G, Lu Z W, Xiang T, Wang L W (2019). Large-scale few-shot learning: Knowledge transfer with class hierarchy. Proceedings of IEEE/CVF Conference on Computer Vision and Pattern Recognition.

[10] Li A X, Zhang K X, Wang L W (2019). Zero-shot fine-grained classification by deep feature learning with semantics. International Journal of Automation and Computing.

[11] Xian Y Q, Lampert C H, Schiele B, Akata Z (2019). Zero-shot learning — A comprehensive evaluation of the good, the bad and the ugly. IEEE Transactions on Pattern Analysis and Machine Intelligence.

[12] Welinder P, Branson S, Mita T, Wah C, Schroff F, Belongie S, Perona P (2010). Caltech-UCSD Birds 200.

[13] Li S C, Chen D P, Liu B, Yu M H, Zhao R (2019). Memory-based neighbourhood embedding for visual recognition. Proceedings of IEEE/CVF International Conference on Computer Vision.

[14] H. X. Yao, X. Wu, Z. Q. Tao, Y. L. Li, B. L. Ding, R. R. Li, Z. H. Li. Automated relational meta-learning. In *Proceedings of the 8th International Conference on Learning Representations*, Addis Ababa, Ethiopia, 2020.

[15] C. Xing, N. Rostamzadeh, B. N. Oreshkin, P. O. Pinheiro. Adaptive cross-modal few-shot learning. In *Proceedings of the 33rd Conference on Neural Information Processing Systems*, Vancouver, Canada, pp. 4848–4858, 2019.

[16] Peng Z M, Li Z C, Zhang J G, Li Y, Qi G J, Tang J H (2019). Few-shot image recognition with knowledge transfer. Proceedings of IEEE/CVF International Conference on Computer Vision.

[17] Debasmit D, George Lee C S (2020). A two-stage approach to few-shot learning for image recognition. IEEE Transactions on Image Processing.

[18] V. G. Satorras, J. B. Estrach. Few-shot learning with graph neural networks. In *Proceedings of the 6th International Conference on Learning Representation*, Vancouver, Canada, 2018.

[19] Kim J, Kim T, Kim S, Yoo C D (2019). Edge-labeling graph neural network for few-shot learning. Proceedings of IEEE/CVF Conference on Computer Vision and Pattern Recognition.

[20] Y. B. Liu, J. Lee, M. Park, S. Kim, E. Yang, S. J. Hwang, Y. Yang. Learning to propagate labels: Transductive propagation network for few-shot learning. In *Proceedings of the 7th International Conference on Learning Representations*, New Orleans, USA, 2019.

[21] Zhou X K, Liang W, Shimizu S, Ma J H, Jin Q (2021). Siamese neural network based few-shot learning for anomaly detection in industrial cyber-physical systems. IEEE Transactions on Industrial Informatics.

[22] Ye H J, Hu H X, Zhan D C (2021). Learning adaptive classifiers synthesis for generalized few-shot learning. International Journal of Computer Vision.

[23] Jamal M A, Qi G J (2019). Task agnostic meta-learning for few-shot learning. Proceedings of IEEE/CVF Conference on Computer Vision and Pattern Recognition.

[24] Obamuyide A, Vlachos A (2019). Model-agnostic meta-learning for relation classification with limited supervision. Proceedings of the 57th Conference of the Association for Computational Linguistics.

[25] Yan S P, Zhang S Y, He X M (2019). A dual attention network with semantic embedding for few-shot learning. Proceedings of the 33rd AAAI Conference on Artificial Intelligence.

[26] S. Ravi, H. Larochelle. Optimization as a model for few-shot learning. In *Proceedings of the 5th International Conference on Learning Representations*, Toulon, France, 2017.

[27] H. X. Yao, X. Wu, Z. Q. Tao, Y. L. Li, B. L. Ding, R. R. Li, Z. H. Li. Automated relational meta-learning. In *Proceedings of 8th International Conference on Learning Representations*, Addis Ababa, Ethiopia, 2020.

[28] A. Nichol, J. Achiam, J. Schulman. On first-order meta-learning algorithms. [Online], Available: https://arxiv.org/abs/1803.02999, 2018.

[29] D. P. Kingma, J. Ba. Adam: A method for stochastic optimization. In *Proceedings of the 3rd International Conference on Learning Representations*, San Diego, USA, 2015.

[30] O. Vinyals, C. Blundell, T. Lillicrap, K. Kavukcuoglu, D. Wierstra. Matching networks for one shot learning. In *Proceedings of the 30th International Conference on Neural Information Processing Systems*, Barcelona, Spain, pp.3637–3645, 2016.

[31] K. R. Allen, E. Shelhamer, H. Shin, J. B. Tenenbaum. Infinite mixture prototypes for few-shot learning. In *Proceedings of 36th International Conference on Machine Learning*, Long Beach, USA, pp. 232–241, 2019.

[32] J. Snell, K. Swersky, R. Zemel. Prototypical networks for few-shot learning. In *Proceedings of the 31st International Conference on Neural Information Processing Systems*, Long Beach, USA, pp. 4080–4090, 2017.

[33] Sung F, Yang Y X, Zhang L, Xiang T, Torr P H S, Hospedales T M (2018). Learning to compare: Relation network for few-shot learning. Proceedings of IEEE/CVF Conference on Computer Vision and Pattern Recognition.

[34] L. Bertinetto, J. F. Henriques, P. H. S. Torr, A. Vedaldi. Meta-learning with differentiable closed-form solvers. In *Proceedings of 7th International Conference on Learning Representations*, New Orleans, USA, 2019.

[35] Hao F S, He F X, Cheng J, Wang L, Cao J Z, Tao D C (2019). Collect and select: Semantic alignment metric learning for few-shot learning. Proceedings of IEEE/CVF International Conference on Computer Vision.

[36] Li A X, Luo T G, Xiang T, Huang W R, Wang L W (2019). Few-shot learning with global class representations. Proceedings of IEEE/CVF International Conference on Computer Vision.

[37] Wu Z Y, Li Y W, Guo L H, Jia K (2019). PARN: Position-aware relation networks for few-shot learning. Proceedings of IEEE/CVF International Conference on Computer Vision.

[38] N. Mishra, M. Rohaninejad, X. Chen, P. Abbeel. A simple neural attentive meta-learner. In *Proceedings of 6th International Conference on Learning Representations*, Vancouver, Canada, 2018.

[39] Munkhdalai T, Yuan X D, Mehri S, Trischler A (2018). Rapid adaptation with conditionally shifted neurons. Proceedings of the 35th International Conference on Machine Learning.

[40] Qiao L M, Shi Y M, Li J, Tian Y H, Huang T J, Wang Y W (2019). Transductive episodic-wise adaptive metric for few-shot learning. Proceedings of IEEE/CVF International Conference on Computer Vision.

[41] W. Y. Chen, Y. C. Liu, Z. Kira, Y. C. F. Wang, J. B. Huang. A closer look at few-shot classification. In *Proceedings of the 7th International Conference on Learning Representations*, New Orleans, USA, 2019.

[42] A. Antoniou, A. J. Storkey. Learning to learn by self-critique. In *Proceedings of the 33rd Conference on Neural Information Processing Systems*, Vancouver, Canada, pp.9936–9946, 2019.

